# Changes in Racial Equity Associated With Participation in the Bundled Payments for Care Improvement Advanced Program

**DOI:** 10.1001/jamanetworkopen.2022.44959

**Published:** 2022-12-05

**Authors:** Gmerice Hammond, E. John Orav, Jie Zheng, Arnold M. Epstein, Karen E. Joynt Maddox

**Affiliations:** 1 Cardiovascular Division, Department of Medicine, Washington University School of Medicine, St. Louis, Missouri; 2 Division of General Internal Medicine, Department of Medicine, Brigham and Women’s Hospital, Boston Massachusetts; 3 Department of Biostatistics, Harvard T. H. Chan School of Public Health, Boston Massachusetts; 4 Department of Health Policy and Management, Harvard T. H. Chan School of Public Health, Boston, Massachusetts; 5 Institute for Public Health at Washington University, St. Louis, Missouri

## Abstract

**Importance:**

The Medicare alternative payment models are designed to incentivize cost reduction and quality improvement, but there are no requirements established for evaluating the outcomes of the Medicare populations.

**Objective:**

To examine whether participation in the Medicare Bundled Payments for Care Improvement Advanced (BPCI-A) program was associated with narrowing or widening of Black and White racial inequities in outcomes and access.

**Design, Setting, and Participants:**

Retrospective cohort alternative payment models on equity and quality for disadvantaged populations were studied between April 6, 2021, and August 28, 2022, in US hospitals. Black and White Medicare beneficiaries admitted for any of the 29 inpatient conditions in the BPCI-A program between January 1, 2017, and September 31, 2019, were included.

**Exposures:**

BPCI-A participation implemented in 2018.

**Main Outcomes and Measures:**

Ninety-day readmission and mortality, healthy days at home, and proportion of Black patients hospitalized. Segmented regression models were used to examine quarterly changes in slopes for each outcome.

**Results:**

The sample included 6 690 336 episodes (6 019 359 White patients, 670 977 Black patients). The population comprised approximately 43% men, 57% women, 17% individuals younger than 65 years, 47% between ages 65 and 80 years, and 36% older than 80 years. Prior to implementation of the BPCI-A program, compared with episodes for White patients, Black patients had higher 90-day readmissions (36.3% vs 29.6%), similar 90-day mortality (12.3% vs 13.3%), and fewer healthy days at home (mean, 68.5 vs 69.5 days). BPCI-A participation was not associated with significant changes in the racial gap in readmissions but was associated with a greater gain in heathy days at home (differences by race, −0.07 days per quarter; 95% CI, −0.12 to −0.01 days per quarter). Among Black patients admitted to BPCI-A hospitals vs controls, healthy days at home increased by 0.09 more days/episode per quarter (95% CI, 0.02-0.17 days/episode per quarter). The proportion of Black patients decreased similarly at BPCI-A and control hospitals.

**Conclusions and Relevance:**

In this cohort study, BPCI-A participation was not associated with improvements in racial inequities in clinical outcomes. Black patients in BPCI-A had a slight gain in healthy days at home; there were no changes in access. The findings of this study suggest that more needs to be done if payment policy reform is going to be part of the efforts to address glaring racial inequities in health care quality and outcomes. These findings support a need for payment policy reform specifically targeting equity-enhancing programs.

## Key Points

**Question:**

Is the Medicare Bundled Payments for Care Improvement Advanced (BPCI-A) program associated with changes in inequities in outcomes or access to care between Black and White patients?

**Findings:**

In this cohort study of 6 690 336 patient episodes, the BPCI-A program was not associated with improvements in existing racial inequities in readmission rates. Black patients in the BPCI-A program had a slight increase in healthy days at home under the program, and there were no significant changes in access to care.

**Meaning:**

The findings of this cohort study suggest that, at the 1-year mark, BPCI-A participation neither narrowed nor worsened racial inequities in clinical outcomes or access.

## Teaser

This cohort study compares outcomes and access to care between Black and White patients following implementation of the Medicare Bundled Payments for Care Improvement–Advanced program.

## Introduction

In efforts to improve outcomes and reduce costs, Medicare and private payers have moved to implement value-based and alternative payment models, novel payment approaches that tie payment not only to the volume of services rendered but also to the quality, outcomes, and/or costs of care provided. The Center for Medicare and Medicaid Innovation (CMMI) has implemented and evaluated more than 50 payment models since its inception in 2011. These evaluations have been critically important, since statutorily, the CMMI can expand its experimental models nationally if they are shown to reduce costs while maintaining or improving quality.^1^

Both the recently released strategic refresh and Healthy People 2030 of CMMI make achieving health equity a central goal.^2,3^ However, there is no current requirement that CMMI measures or assesses equity in their programmatic evaluations. That does not negate the importance of determining whether these programs impact equity as the Centers for Medicare & Medicaid Services decides whether to continue programs and how to modify them to be equity-enhancing. The rationale for doing so in regard to racial equity is strong; due to social and structural determinants of health and racism, there are striking racial inequities in health outcomes, and many are widening over time.^4,5,6,7,8,9^ Addressing these inequities is a national imperative. However, to our knowledge, performing a racial equity assessment when considering whether to continue or to scale novel payment models nationally has not been pursued.

There are at least 3 questions that should be asked when examining the racial equity outcomes of payment models. First, is the model associated with a narrowing or a widening of existing gaps in quality or health outcomes, meaning in relative terms, did quality or outcomes improve or worsen for Black patients compared with White patients? Second, in absolute terms, is the model associated with changes in quality or outcomes for Black patients? Third, is the model associated with any changes in access to care for Black patients? Given prior research reporting that Black patients have significant unmet clinical needs and worse access to care and therefore lower costs than would be optimal in some settings,^10^ we did not consider costs to be an equity target.

Using this paradigm, we examined the equity impact of a recently implemented alternative payment model focused on episode-based payments for hospitalized patients, the Medicare Bundled Payments for Care Improvement Advanced (BPCI-A) program. The BPCI-A program, introduced in 2018, holds participating hospitals accountable for a 90-day episode of care triggered by a hospitalization.^11^ Overall, the program saw participation of approximately 10% of US hospitals. The goals of the program were to incentivize care redesign and better care coordination. Under the BPCI-A program, cost targets for episodes were established for participants. A portion of any expenditures that exceeded targets were penalties that had to be paid back to Medicare, while a portion of cost savings were kept by participants. Prior studies reported that the BPCI-A program was associated with reductions in Medicare payments per episode.^12^ We focused on the 3 questions described above and applied them to the BPCI-A program to determine what the equity implications were at the 1-year mark as an equity safety check on the BPCI-A program.

## Methods

### Data Source and Study Sample

This study was approved by the Office of Human Research Protection at Washington University School of Medicine. The requirement for informed consent was waived due to the deidentified nature of the data. The report follows the Strengthening the Reporting of Observational Studies in Epidemiology (STROBE) reporting guideline for cohort studies. Data analysis occurred between April 6, 2021, and August 28, 2022.

A complete list of BPCI-A participant hospitals is publicly available.^13^ Participation began in October 2018; an additional wave of participants joined in January 2020. Participants joining in the initial wave were the intervention group for this study, and hospitals that joined in January 2020 were excluded from analyses because the program was put on hold in early 2020 due to the COVID-19 pandemic. All remaining US hospitals paid under the inpatient prospective payment system were included as controls. We used American Hospital Association data^14^ and Area Health Resources File^15^ data to characterize hospitals and geographic markets. Six of 832 BPCI-A participants and 182 of 2198 comparison hospitals did not match to American Hospital Association or Area Health Resources data and were excluded from the analyses.

To obtain information on patients and health outcomes, we analyzed Medicare claims data from January 1, 2017, to December 31, 2019, capturing all episodes initiated on or before September 30, 2019. October 2018 was considered the beginning of the postintervention period. Patients were included if they were admitted for any of the 29 qualifying conditions defined by BPCI-A eligibility criteria (eTable in the Supplement). We only included patients who were continuously enrolled in Medicare Parts A and B during their episode of care and the year prior, and we excluded those with Medicare eligibility due to end-stage renal disease per BPCI-A program specifications. All episodes were for fee-for-service beneficiaries since the BPCI-A program only includes fee-for-service beneficiaries. The Medicare Virtual Research Data Center was used to access Medicare data.^16^

### Missing Data

In the broader sample of patients admitted to US acute care hospitals with any of the BPCI-A program conditions, 0.81% of patients were classified as unknown race and could not be appropriately included in our analytic sample. At the hospital level, missing data that made it impossible to link a hospital with its characteristics or location led to the exclusion of an additional 0.03% of patients. Our analytic sample had no further missing data.

### Covariates

Our primary variable was patient race, which was obtained from Medicare enrollment data. Medicare defines race and ethnicity as American Indian/Alaska Native, Asian/Pacific Islander, Black, Hispanic, White, or other. Due to small numbers of patients in the racial groups other than Black and White, we limited the study to Black and White patients. Other important covariates included measures of social risk (Medicaid enrollment status as a marker for poverty) as well as clinical comorbidities, defined using the Medicare Chronic Conditions Data Warehouse. We also obtained information on hospitals from the 2019 American Hospital Association database, and on communities from the 2019 American Community Survey and Area Health Resource File. Market characteristics were evaluated at the county level for 2017 and included the following: proportion of the population older than 65 years, median income, percent Medicare Advantage, number of skilled nursing facilities per 10 000 patients, number of rehabilitation hospitals, market share, and Herfindahl-Herschman Index.

### Outcomes

Key outcomes examined included quality, measured via 90-day readmission rate and 90-day mortality rate, following Centers for Medicare & Medicaid Services specifications, as well as the number of healthy days at home, and access to care, which was assessed by determining the proportion of episodes for Black patients before and after program participation.

### Statistical Analysis

Greater detail is provided in the eMethods in the Supplement. Briefly, we first compared episode, hospital, and market characteristics between BPCI-A participating hospitals and nonparticipants. We tested for parallel trends in our key outcomes and found that this assumption was violated, thereby making it inappropriate to use standard difference-in-differences models. Thus, as has been done previously,^12^ we used a segmented regression model with a control group^17,18^ to examine quarterly changes in slopes for each outcome during the baseline vs intervention period. The change in slope for Black BPCI-A program participants was compared with White participants to see whether the program performed equally for Black and White participants; the change in slope for Black nonparticipants was compared with White nonparticipants, and these 2 changes in slope were compared. To address our second question (ie, whether the model was associated with improvements in quality or outcomes for Black patients), differences in the slope change of outcomes between Black patients hospitalized at BPCI-A program participant and control hospitals were compared. An analogous final model compared the slope change in the proportion of Black patients in BPCI-A vs non-BPCI-A program hospitals to determine whether there had been a change in access. A marginal, generalized estimating equation–based linear model was run for each outcome (the GENMOD procedure in SAS, version 9.4; SAS Institute Inc) based on episode-level outcome data. The model included hospital fixed effects to account for correlation within hospitals over time and robust SEs. Covariates included indicator variables for diagnosis-related groups, patient age and sex, Medicaid, disability, individual patient-level Chronic Conditions Data Warehouse comorbidities, and community characteristics. Linear probability models were used for all outcomes for interpretability. The 2-sided, unpaired significance threshold was *P* = .05.

## Results

### Episode Characteristics

The final sample included 6 019 359 White patients and 670 977 Black patients (eFigure in the Supplement). The population comprised approximately 43% men, 57% women, 17% individuals younger than 65 years, 47% between ages 65 and 80 years, and 36% older than 80 years There were 1 461 222 episodes among BPCI-A participating hospitals (n = 826), of which 88.8% were for White patients (56.0% women) and 11.2% for Black patients (58.2% women). There were 5 229 114 episodes at control hospitals (n = 2016); 90.3% were for White patients (56.2% women) and 9.7% were for Black patients (57.4% women). At both BPCI-A and control hospitals, compared with episodes for White patients, those for Black patients were more likely to be for individuals younger than 65 years, dually insured with Medicare and Medicaid, and qualified for Medicare on the basis of a disability (Table 1). At both BPCI-A participating hospitals and control hospitals, episodes for Black patients were more likely to be at major teaching hospitals than were episodes for White patients (Table 1). Black patients’ episodes tended to be in hospitals in counties with a higher population, lower median income, higher postacute care supply, and less market consolidation, as defined by the Herfindahl-Hirschman Index.

**Table 1.** Episode Characteristics of All 29 Condition Patient Dyads

Variable	BPCI-A participants (n = 1 461 222)			Non-BPCI-A participants (n = 5 229 114)		
	White	Black	SMD^a^	White	Black	SMD^a^
No. (%)	1 297 335 (88.8)	163 887 (11.2)		4 722 024 (90.3)	507 090 (9.7)	
No. of episodes per quarter, mean	22.0	4.9	−0.805	12.8	3.7	−0.536
Age (%), y						
<65	9.2	23.7	0.476	10.5	23.9	0.421
65-80	46.2	45.8	−0.008	49.0	47.0	−0.04
≥80	44.7	30.5	−0.288	40.5	29.1	−0.235
Sex, %						
Male	44.0	41.8	0.045	43.8	42.6	0.025
Female	56.0	58.2	0.045	56.2	57.4	0.025
Medicaid, %	21.0	53.1	0.767	22.8	52.4	0.692
Disabled, %	21.6	43.9	0.53	23.9	44.3	0.469
Total No. of CCWs	6.21	6.60	0.114	5.78	6.08	0.09
Complications, %						
Major complication	47.4	53.7	0.126	40.8	44.6	0.079
Minor complication	30.3	22.5	−0.173	34.5	26.8	−0.165
No complication	22.1	23.7	0.038	24.0	28.0	0.093
With outlier payments, %	1.5	1.7	0.016	5.3	12.3	0.294
Hospital profit status, %						
For profit	23.3	23.8	0.011	11.7	12.0	0.012
Not for profit	71.0	68.3	−0.059	75.0	70.2	−0.111
Public	5.6	7.9	0.096	13.3	17.8	0.13
Hospital size, %						
Small	4.9	3.4	−0.071	15.6	9.8	−0.164
Medium	60.2	56.2	−0.083	55.6	48.7	−0.14
Large	34.7	40.3	0.116	28.7	41.5	0.28
Hospital teaching status, %						
Major teaching	18.1	26.0	0.203	13.5	25.0	0.327
Minor teaching	40.2	37.3	−0.059	33.5	32.1	−0.03
Nonteaching	41.6	36.5	−0.104	53.0	42.9	−0.203
Hospital location. %						
Rural	1.0	0.7	−0.031	3.4	3.5	0.008
Hospital region, %						
Northeast	22.0	18.6	−0.083	19.9	12.9	−0.178
Midwest	25.3	24.7	−0.013	23.9	13.5	−0.246
South	37.9	46.5	0.176	41.9	67.3	0.517
West	14.6	10.0	−0.132	14.3	6.3	−0.236
Hospital in a system, %	35.9	33.2	−0.055	19.5	16.8	−0.068
County level^b^						
Individuals aged ≥65 y	1 142 349	1 630 575	0.272	594 487	(874 325	0.224
Median income, $	62 814	59 411	−0.214	60 006	58 629	−0.083
Medicare Advantage, %	31.8	32.8	0.09	28.9	(28.9	−0.002
SNF beds/10 000	5400.9	7582.4	0.271	2851.4	4317.8	0.272
No. rehabilitation hospitals, mean	0.95	1.2	0.172	0.53	0.71	0.181
Market share, %	0.42	0.35	−0.218	0.58	0.48	−0.292
Herfindahl-Herschman Index	0.17	0.14	−0.223	0.21	0.18	−0.152

Abbreviations: BPCI-A, Bundled Payments for Care Improvement–Advanced; CCW, Chronic Conditions Data Warehouse; SMD, standard mean difference; SNF, skilled nursing facility.

^a^ The SMD is a summary statistic that represents the number of SD by which the 2 groups differ and is a way of normalizing the differences across variables that might have very different units of measurement. Values less than 0.1 suggest high comparability between groups.

^b^ All county-level variables are from 2017.

### Changes in Outcomes by Race in BPCI-A Participants vs Controls

**Figure 1.**
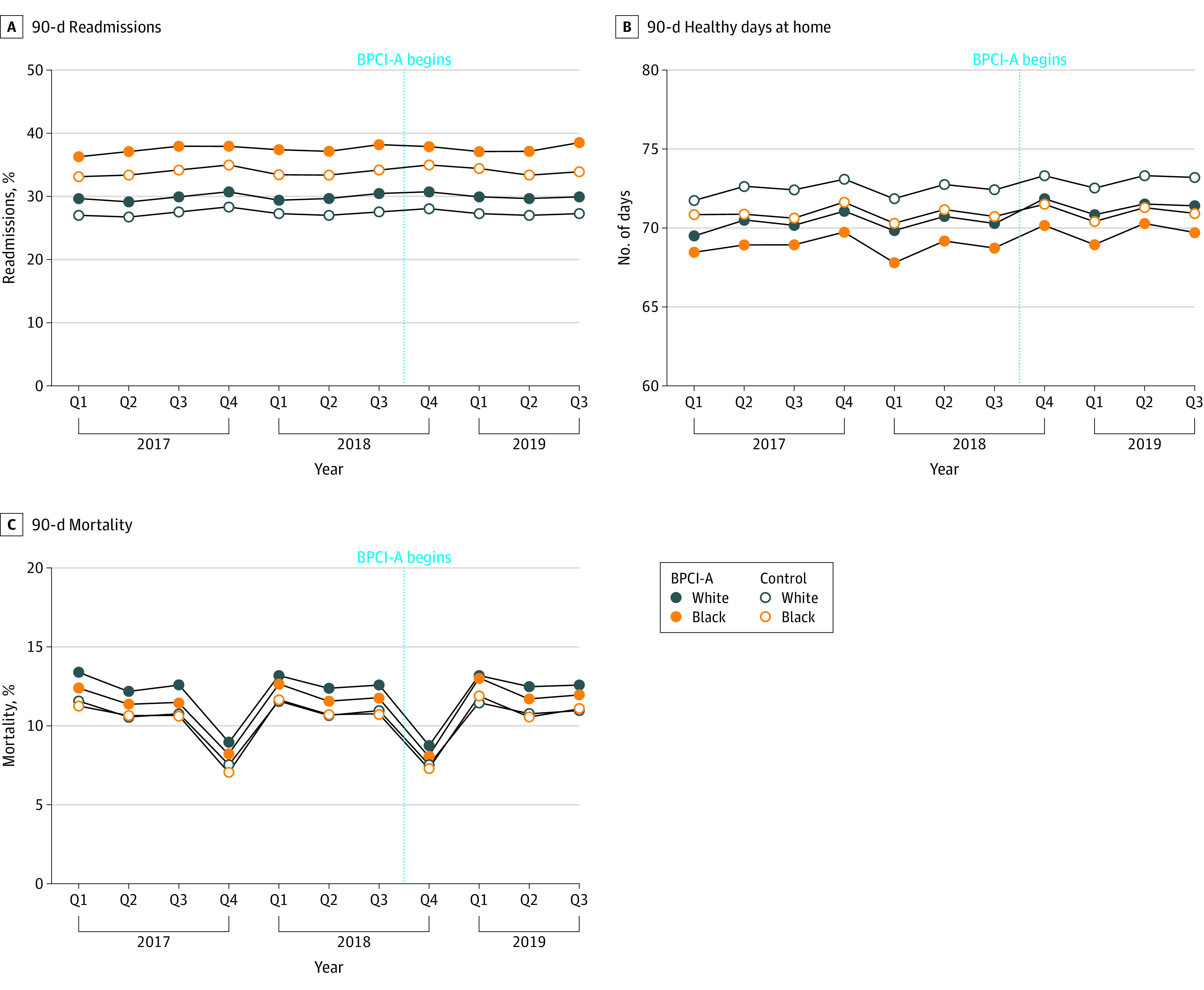
Outcomes by Race Changes in outcomes for readmissions (A), healthy days at home (B), and mortality (C).

At both BPCI-A program participating hospitals and controls, Black patients had higher 90-day readmission rates vs White patients at baseline (BPCI-A hospitals, 36.3% vs 29.6%; controls, 33.1% vs 27.1%) (Figure 1). At BPCI-A hospitals, among White patients, readmissions were increasing at 0.12% per quarter before participation, and at 0.05% per quarter during the intervention, for a difference of −0.07% per quarter (Table 2). Among Black patients, readmission rates were increasing at 0.21% per quarter before participation, and 0.09% per quarter during the intervention, for a difference of −0.12% per quarter, and a difference in differences by race of 0.05% per quarter (95% CI, −0.05% to 0.15%). Similarly, in the control group, readmission rates for both Black and White patients increased more slowly during the intervention, with a difference in differences by race of −0.01% per quarter (95% CI, −0.07% to 0.05%). The 3-way interaction term was nonsignificant (*P* = .27), suggesting that BPCI-A was not associated with a significant change in the racial gap in this outcome compared with controls.

**Table 2.** Adjusted Differential Changes by Race in Outcomes Associated with BPCI-A^a^

Outcome	Group	Race	Baseline value	Quarterly change		Difference in quarterly change (post minus pre)	Difference in differences		95% CI	*P* value for 3-way interaction^b^
				Preintervention	Postintervention		White minus Black	BPCI-A minus control		
90-d Readmission rate, %	BPCI-A	Black	36.3	0.21	0.09	−0.12	NA	NA		.27
		White	29.6	0.12	0.05	−0.07	0.05	NA	−0.04 to 0.15	
	Control	Black	33.1	0.12	0.08	−0.04	NA	NA		
		White	27.1	0.06	0.01	−0.05	−0.01	NA	−0.06 to 0.04	
90-d Mortality rate, %	BPCI-A	Black	12.3	0.02	−0.04	−0.06	NA	NA		.68
		White	13.4	−0.02	−0.06	−0.04	0.02	NA	−0.05 to 0.08	
	Control	Black	11.3	0.01	−0.05	−0.05	NA	NA		
		White	11.5	0.02	−0.03	−0.05	0.00	NA	−0.03 to 0.04	
Healthy days at home	BPCI-A	Black	68.5	0.01	0.14	0.13	NA	NA		.01
		White	69.5	0.13	0.19	0.06	−0.07	NA	−0.12 to 0.01	
	Control	Black	70.8	0.04	0.07	0.03	NA	NA		
		White	71.7	0.08	0.12	0.04	0.01	NA	−0.02 to 0.04	
90-d Readmission rate, %	BPCI-A	Black	36.3	0.21	0.09	−0.12	NA	−0.07	−0.19 to 0.04	.19
	Control	Black	33.1	0.12	0.08	−0.04	NA	NA	NA	
90-d Mortality rate, %	BPCI-A	Black	12.3	0.02	−0.04	−0.06	NA	−0.01	−0.09 to 0.07	.81
	Control	Black	11.3	0.01	−0.05	−0.05	NA	NA	NA	
Healthy days at home, %	BPCI-A	Black	68.5	0.01	0.14	0.13	NA	0.10	0.02 to 0.17	.01
	Control	Black	70.8	0.04	0.07	0.03	NA	NA	NA	

Abbreviations: BPCI-A, Bundled Payments for Care Improvement–Advanced; NA, not applicable.

^a^ All outcomes were adjusted for age, sex, Medicaid enrollment, disability, Chronic Conditions Data Warehouse comorbidities, diagnosis-related group, and hospital profit status, urban vs rural location, teaching status, and region.

^b^ *P* values for 3-way interaction tests whether the change in the BPCI-A group from preintervention to postintervention for Black vs White patients is different than the change in the control group from preintervention to postintervention for Black vs White patients.

At both BPCI-A participating hospitals and controls, Black patients had similar 90-day mortality rates to White patients at baseline (BPCI-A hospitals, 12.3% vs 13.4%; controls, 11.3% vs 11.5%) (Figure 1). At BPCI-A hospitals, among White patients, mortality rates were flat at −0.02% per quarter before participation but decreased at −0.06s% per quarter during the intervention, for a difference of −0.04% per quarter (Table 2). Among Black patients, mortality rates were flat at 0.02% per quarter before participation and −0.04% per quarter during the intervention, for a difference of −0.06% per quarter and a difference in differences of 0.02% per quarter (95% CI, −0.05% to 0.08% per quarter). Similarly, in the control group, mortality rates for both Black and White patients were decreasing to a greater degree during the intervention compared with the preparticipation period, with a difference in differences by race of 0.00% per quarter (95% CI, −0.03% to 0.04% per quarter). The 3-way interaction term was nonsignificant (*P* = .68), suggesting that the BPCI-A program was not associated with a significant change in the racial gap in this outcome compared with controls.

At both BPCI-A participating hospitals and controls, Black patients had fewer healthy days at home than White patients at baseline (BPCI-A hospitals; mean, 68.5 vs 69.5 days; controls, 70.8 vs 71.7 days) (Figure 1). At BPCI-A hospitals, among White patients, healthy days at home were increasing at 0.13 days per quarter before participation and at 0.19 days per quarter during the intervention, for a difference of 0.06 days per quarter (Table 2). Among Black patients, healthy days at home were flat at 0.01 days per quarter before participation but increased at 0.14 days per quarter during the intervention, for a difference of 0.13 days per quarter and a difference in differences by race of −0.07 days per quarter (95% CI, −0.12 to −0.01 days per quarter) or a total of 11 472 days for the entire cohort (0.07 × 169 887 episodes). In the control group, healthy days at home for both Black and White patients were increasing to a greater degree during the intervention compared with the preintervention period, for a difference in differences by race of 0.01 days per quarter (95% CI, −0.02 to 0.04 days per quarter). The 3-way interaction term was significant (*P* = .01), suggesting that BPCI-A was associated with a small but statistically significant narrowing in the racial gap in this outcome compared with controls.

### Outcomes for Black Patients at BPCI-A Compared With Control Hospitals

There were no differential changes when comparing Black patients at BPCI-A hospitals with Black patients at control hospitals in 90-day readmission or mortality rates. However, healthy days at home increased more among Black patients at BPCI-A participating hospitals vs control hospitals: 0.13 vs 0.03 days/quarter, with difference in differences by BPCI-A participation 0.09 days per quarter (95% CI, 0.02-0.17 days/episode per quarter), or a total of 15 289 days for the entire cohort (0.09 × 169 887 episodes) (Table 2**)**.

### Access to Care for Black Patients at BPCI-A Compared With Control Hospitals

**Figure 2.**
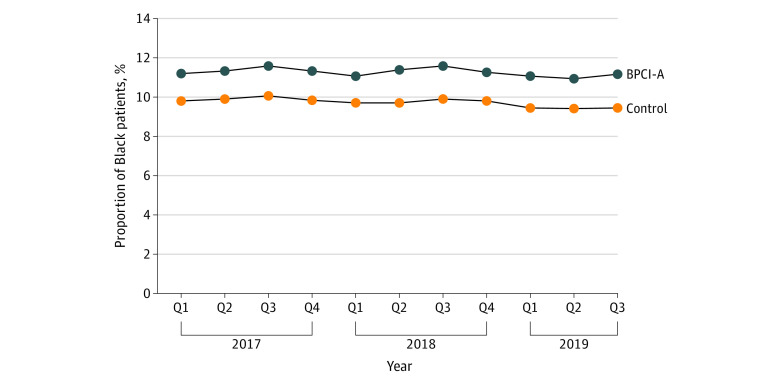
Proportion of Black Patients by Bundled Payments for Care Improvement–Advanced (BPCI-A) Program Group Q indicates quarter.

Black patients represented a higher proportion of patients at BPCI-A hospitals vs control hospitals at baseline (11.2% vs 9.8%) (Figure 2). At BPCI-A hospitals, this proportion was increasing at 0.02% per quarter before participation but decreased at −0.02% per quarter during the intervention, for a difference of −0.04% per quarter. In the control group, the proportion of Black patients was decreasing at −0.02% per quarter in the preintervention period and at −0.04% per quarter during the intervention period, for a difference of −0.02% per quarter and a difference in differences by BPCI-A participation of −0.02% per quarter (95% CI, −0.05% to 0.01% per quarter), suggesting no differential change in the proportion of Black patients at BPCI-A hospitals vs controls.

## Discussion

At baseline, Black beneficiaries had higher readmission rates, similar mortality rates, and fewer healthy days at home than their White counterparts. Participation in the BPCI-A program was associated with a small but statistically significant reduction in racial inequities in healthy days at home, and Black beneficiaries in BPCI-A hospitals had increases in healthy days at home compared with Black beneficiaries in control hospitals during the study period. There was no differential change in the proportion of Black patients admitted to BPCI-A hospitals compared with control hospitals. To our knowledge, these are the first data examining quality and equity of care by race for the BPCI-A program.

Participation in the BPCI-A program was not associated with either a narrowing or a widening of existing racial inequities in readmissions. Although the program did not incent equity per se, many had hoped that the BPCI-A program emphasis on care coordination, standardization of care, and possibly a resultant greater attention to social determinants of health and continuity would disproportionately benefit groups that have historically and systematically been recipients of poorly coordinated and inferior care, such as racially minoritized patients. One possibility for why there was only inconsistent benefit in Black compared with White patients is that hospitals may not have focused on race as a risk factor and may not have developed programs specific to Black beneficiaries as part of care redesign. If Black beneficiaries have different needs or gaps in care than White ones, generalized interventions may not have been successful in broadly reducing inequities. Even if hospitals had programs aimed at high-risk groups, implementation may not have been optimal; intention to implement a program does not ensure effective execution of care redesign or other strategies. Another possibility is that these kinds of changes may take more time to manifest in measurable differences.

We also found that healthy days at home increased slightly for Black patients compared with White patients and among Black patients at BPCI-A hospitals compared with Black patients at control hospitals. In addition to its effects on equity, understanding a program’s absolute effect on Black patients is an important part of program evaluation and could drive precision policy. If there are benefits for Black patients, key focused elements of these programs could be scaled more broadly. Only examining outcomes overall and not stratifying by race would potentially miss benefits accruing to Black patients and thereby miss an opportunity to learn about important targeted care improvement strategies.

We did not find any negative outcomes of the BPCI-A program on access to care, as measured by the proportion of episodes at each hospital that were for Black patients. Although some of the conditions included in the BPCI-A program are elective, such as joint replacement, many are not, such as heart failure, stroke, and sepsis. It is possible that the breadth of the program reduces its negative selection effects, since many hospitals chose both elective and nonelective conditions for participation.

### Limitations

There are limitations to our study. We used claims data and were therefore limited in our ability to ascertain comorbidities; if Black patients have less access to outpatient and long-term care than White patients, it is possible that their comorbidities are undercaptured in these data.^10^ Race as a variable is limited in and of itself; not only does it lack precision in describing individuals, but in this setting it is being used as an imperfect proxy for racism. We do not have access to data on patient-reported outcomes, quality of life, functional status, or other more subtle outcomes that might be affected under these programs and represent important areas for future study. Our measure of access has 2 specific limitations: because there is no true community-based denominator of patients who need specific elective procedures, we can only measure the proportion of patients who are Black among those who receive elective procedures, which is sensitive to volume changes in either group. We also recognize that reducing episode volume for urgent admissions, such as strokes, is potentially good. Therefore, all analyses of access should be considered exploratory.^19^ We cannot fully account for other concurrent payment policies, such as accountable care organizations or physician group participation in the BPCI-A program. Our follow-up time was limited, and longer, prospective evaluations should continue to track the association of the BPCI-A program and other payment models with outcomes and equity for Black beneficiaries going forward.

## Conclusions

In this cohort study of the BPCI-A program, participation in the program was not associated with improvements in existing racial inequities in 90-day mortality or 90-day readmission. Black patients in the BPCI-A program had a slight gain in healthy days at home under the program, and there were no significant changes in access to care. While there was no evidence of adverse association with equity in this study, our findings did not show meaningful improvement in existing inequities in quality and outcomes among participants in this program. Although it is important and necessary that payment policies avoid harm, it is insufficient. These findings support a need for payment policy reform specifically targeting equity-enhancing programs. More intentional and targeted efforts need to be made if payment policy reform is going to contribute to reducing glaring racial inequities in health care quality and outcomes. In addition, we suggest that quality be redefined to include equity and that equity be assessed across at least these 3 dimensions: whether the model leads to widening or narrowing of inequities in outcomes for disadvantaged populations, whether the model results in better outcomes for such populations, and whether the model results in any changes in access to care. Ongoing, prospective evaluation is needed to ensure payment innovations improve rather than worsen equity.

## Notes

Supplement. **eMethods.** Detailed Methods **eFigure.** Exclusion/Inclusion Criteria Flow Chart **eTable.** Number of BPCI-A Hospital Episodes and Conditions
eReferences
