# Supplementary material for: Changes in Racial Equity Associated With Participation in the Bundled Payments for Care Improvement Advanced Program
Source: JAMA Netw Open. 2022 Dec 5;5(12):e2244959. doi: 10.1001/jamanetworkopen.2022.44959 (PMC9855294; doi:10.1001/jamanetworkopen.2022.44959)
Supplement: Supplement. — eMethods. Detailed Methods eFigure. Exclusion/Inclusion Criteria Flow Chart eTable. Number of BPCI-A Hospital Episodes and Conditions eReferences [file jamanetwopen-e2244959-s001.pdf]

Supplementary Online Content

Hammond G, Orav EJ, Zheng J, Epstein AM, Joynt Maddox KE. Changes in racial equity associated with participation in the Bundled Payments for Care Improvement Advanced program. *JAMA Netw Open*. 2022;5(12):e2244959.
doi:10.1001/jamanetworkopen.2022.44959

eMethods. Detailed Methods

eFigure. Exclusion/Inclusion Criteria Flow Chart

eTable. Number of BPCI-A Hospital Episodes and Conditions

eReferences

This supplementary material has been provided by the authors to give readers additional information about their work.

© 2022 Hammond G et al. *JAMA Network Open.*

eMethods. Detailed Methods

We first compared episode, hospital, and market characteristics between BPCI-A participating hospitals and non-participants. We tested for parallel trends in our key outcomes, and found that this assumption was violated, thereby making it inappropriate to use standard difference in differences models.

Thus, as has been done previously,[1] we then used a segmented regression model,[2,3] modified to include a control group, to examine quarterly changes in slopes for each outcome during the baseline versus the intervention period. The change-in-slope for Black BPCI-A participants was compared to the change-in-slope for White participants to see if the program performed equally for Black and White participants; then, the change-in-slope for Black non-participants was compared to the change-in-slope for White non-participants to see if there were comparable time trends for Black and White patients outside of the program. To address our second question, whether the model was associated with improvements in quality or outcomes for Black patients, we compared differences in the slope change of outcomes between Black patients hospitalized at BPCI-A participant and control hospitals to see if the BPCI-A program was associated with improved performance for Black beneficiaries. An analogous final model was run with patient racial group (Black versus White) as the outcome, rather than a covariate. This model allowed us to compare the slope change in the proportion of Black patients who were admitted pre- versus post-intervention in BPCI-A versus non-BPCI-A hospitals to determine whether there had been a change in access. A marginal, generalized estimating equation based linear model was run for each outcome (the GENMOD procedure in SAS 9.4) based on episode-level outcome data. The model included hospital fixed effects to account for correlation within hospitals over time, and robust standard errors. Covariates included time (pre-post intervention) and indicator variables for DRGs, patient age, sex, Medicaid, disability, individual patient-level CCW comorbidities, and community characteristics including proportion of the population over 65 years of age, median income, % Medicare Advantage, number of skilled nursing facilities/10,000, number of rehabilitation hospitals, market share, and Herfindahl-Herschman Index. Linear probability models were used for all outcomes for interpretability.

The model is as follows, using healthy days at home as an example:

> Expected healthy days at home = Intercept + Time1 + Time2 + BPCI-A + Black + Time1*BPCI-A + Time2*BPCI-A + Time1*BPCI-A*Black + Time2*BPCI-A*Black + Age + Sex + Medicaid + Disability + Month (1-12) + DRG (1-101) + CCW (1-27) + Hospital Dummy (hospital fixed effects) + proportion of the population over 65 years of age + median county income + % Medicare Advantage + number of skilled nursing facilities/10,000 population + number of rehabilitation hospitals + market share + Herfindahl-Herschman Index

Where the p-value associated with the Time2*BPCI-A*Black term indicates whether there was a differential change in slope for the outcome in question among Black compared to White patients in BPCI-A compared to control hospitals.

© 2022 Hammond G et al. *JAMA Network Open.*

eFigure: Exclusion/Inclusion Criteria Flow Chart

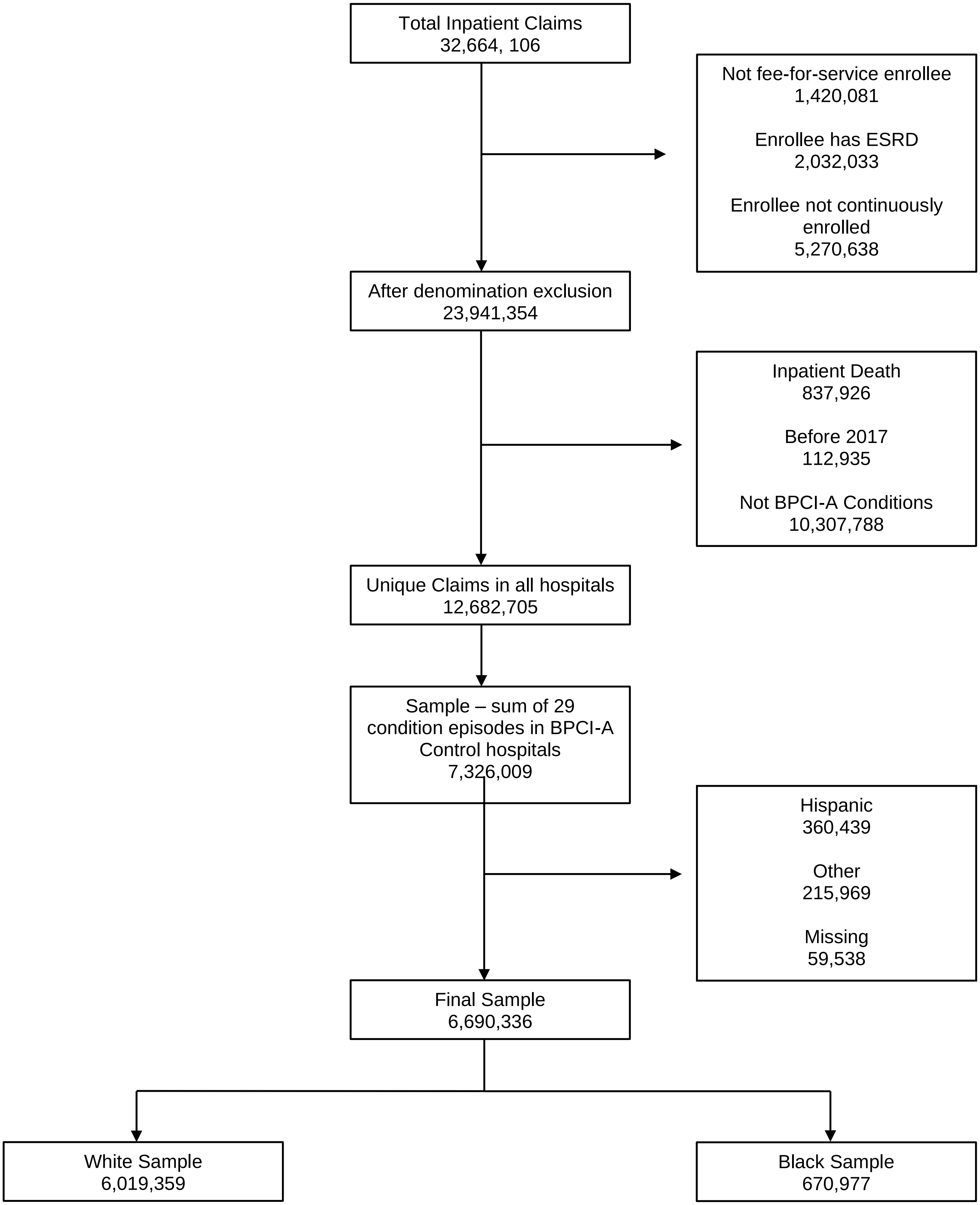

© 2022 Hammond G et al. *JAMA Network Open.*

eTable: Number of BPCI-A Hospital Episodes and Conditions

Conditions	N	Percent	Cumulative Frequency	Cumulative Percent
ACUTE MYOCARDIAL INFARCTION	250	4.5	250	4.5
BACK & NECK EXCEPT SPINAL FUSION	92	1.65	342	6.15
CARDIAC ARRHYTHMIA	357	6.42	699	12.57
CARDIAC DEFIBRILLATOR	50	0.9	749	13.47
CARDIAC VALVE	36	0.65	785	14.12
CELLULITIS	187	3.36	972	17.48
CERVICAL SPINAL FUSION	95	1.71	1067	19.19
CHRONIC OBSTRUCTIVE PULMONARY DISEASE	323	5.81	1390	25
COMBINED ANTERIOR POSTERIOR SPINAL FUSION	12	0.22	1402	25.22
CONGESTIVE HEART FAILURE	489	8.79	1891	34.01
CORONARY ARTERY BYPASS GRAFT	110	1.98	2001	35.99
DISORDERS OF LIVER EXCEPT MALIGNANCY	58	1.04	2059	37.03
DOUBLE JOINT REPLACEMENT OF THE LOWER EXTREMITY	13	0.23	2072	37.27
FRACTURES OF THE FEMUR AND HIP OR PELVIS	76	1.37	2148	38.63
GASTROINTESTINAL HEMORRHAGE	208	3.74	2356	42.37
GASTROINTESTINAL OBSTRUCTION	165	2.97	2521	45.34
HIP & FEMUR PROCEDURES EXCEPT MAJOR JOINT	246	4.42	2767	49.77
LOWER EXTREMITY AND HUMERUS PROCEDURE EXCEPT HIP	103	1.85	2870	51.62
MAJOR BOWEL PROCEDURE	103	1.85	2973	53.47
MAJOR JOINT REPLACEMENT OF THE LOWER EXTREMITY	268	4.82	3241	58.29
MAJOR JOINT REPLACEMENT OF THE UPPER EXTREMITY	76	1.37	3317	59.66
PACEMAKER	125	2.25	3442	61.91
PERCUTANEOUS CORONARY INTERVENTION	198	3.56	3640	65.47
RENAL FAILURE	292	5.25	3932	70.72
SEPSIS	436	7.84	4368	78.56
SIMPLE PNEUMONIA AND RESPIRATORY INFECTIONS	386	6.94	4754	85.5
SPINAL FUSION (NON-CERVICAL)	125	2.25	4879	87.75
STROKE	332	5.97	5211	93.72
URINARY TRACT INFECTION	349	6.28	5560	100

© 2022 Hammond G et al. *JAMA Network Open.*

eReferences:

1. Joynt Maddox KE, Orav EJ, Zheng J, Epstein AM. Year 1 of the Bundled Payments for Care Improvement-Advanced Model. *N Engl J Med*. Aug 12 2021;385(7):618-627. doi:10.1056/NEJMsa2033678
2. Mascha EJ, Sessler DI. Segmented Regression and Difference-in-Difference Methods: Assessing the Impact of Systemic Changes in Health Care. *Anesthesia & Analgesia*. 2019;129(2):618-633. doi:10.1213/ane.0000000000004153
3. Lopez Bernal J, Cummins S, Gasparrini A. Interrupted time series regression for the evaluation of public health interventions: a tutorial. *Int J Epidemiol*. 2016;46(1):348-355.

© 2022 Hammond G et al. *JAMA Network Open.*
